# Relationship between Serum Indirect Bilirubin Level and Insulin Sensitivity: Results from Two Independent Cohorts of Obese Patients with Impaired Glucose Regulation and Type 2 Diabetes Mellitus in China

**DOI:** 10.1155/2020/5681296

**Published:** 2020-07-29

**Authors:** Fan Zhang, Wei Guan, Zhenzhen Fu, Li Zhou, Wen Guo, Yizhe Ma, Yingyun Gong, Wanzi Jiang, Hui Liang, Hongwen Zhou

**Affiliations:** ^1^Department of Endocrinology and Metabolism, The First Affiliated Hospital of Nanjing Medical University, Nanjing 210029, China; ^2^Department of Endocrinology, The Third People's Hospital of Changzhou, Changzhou 213001, China; ^3^Department of General Surgery, The First Affiliated Hospital of Nanjing Medical University, Nanjing 210029, China; ^4^Department of Health Promotion Center, The First Affiliated Hospital of Nanjing Medical University, Nanjing 210029, China

## Abstract

**Background:**

Serum bilirubin is an endogenous antioxidant that has protective effects against obesity-related metabolic diseases.

**Objectives:**

This study aimed to evaluate the characteristics of total bilirubin (TBIL), direct bilirubin (DBIL), and indirect bilirubin (IBIL) and their relationships with insulin sensitivity in obese patients with impaired glucose regulation and type 2 diabetes mellitus (IGR/T2DM) in China. *Patients and Methods*. Cohort 1 comprised obese patients (*n* = 71) was divided into the IGR/T2DM group (*n* = 38, obesity with IGR/T2DM) and control group (*n* = 33, obesity without IGR/T2DM). Insulin sensitivity was evaluated using the hyperinsulinemic-euglycemic clamp technique (HEC) with glucose disposal rate (GDR, *M* value). Cohort 2 comprised obese patients with IGR/T2DM who underwent metabolic surgery (*n* *=* 109) as complementary to cohort 1. Insulin sensitivity was evaluated with the Matsuda Index and homeostatic model assessment of insulin sensitivity (HOMA-IS).

**Results:**

In cohort 1, TBIL, DBIL, and IBIL were higher within the physiological range in the IGR/T2DM group compared with the control group; IBIL was positively correlated with *M* value (*r* *=* 0.342, *p*=0.044) in the IGR/T2DM group, and multivariate logistic regression showed that IBIL might be independent protective factors against insulin resistance (odds ratio (OR) = 0.602; 95% confidence interval (CI): 0.413–0.878; *p*=0.008). In cohort 2, at 1 month after metabolic surgery, serum bilirubin levels (TBIL, DBIL, and IBIL) increased, and the percentage change in IBIL was positively correlated with the change of the Matsuda Index (*r* = 0.195, *p*=0.045).

**Conclusions:**

The relationships between different types of bilirubin and insulin sensitivity varied. Serum indirect bilirubin might be a protective factor that enhances insulin sensitivity.

## 1. Introduction

Obesity remains one of the most prominent risk factors for disease widely prevalent across the world. It is associated with chronic diseases including type 2 diabetes mellitus (T2DM), cardiovascular disease, and nonalcoholic fatty liver disease. A study published in 2017 reported that China had one of the fastest growths in obesity [1]. Insulin resistance is strongly associated with obesity-related impaired glucose regulation (IGR) and T2DM, and oxidative stress and inflammation are potential etiological factors.

Bilirubin is the final product of hemoglobin catabolism in the systemic circulation. In 1987, Stocker et al. [2] found that bilirubin played a beneficial role as a physiological antioxidant. Recent studies have shown that a mild increase in the bilirubin level within the physiological range has a protective effect against obesity-related metabolic diseases [3–5]. The mechanism of the association between these metabolic diseases and bilirubin may be related to the insulin resistance status. Bilirubin could be involved in the signal transduction of the insulin signaling pathway, enhancing insulin sensitivity by reducing oxidative stress and inflammatory responses [6, 7].

Total bilirubin (TBIL) is composed of about 80% indirect bilirubin (IBIL) and 20% direct bilirubin (DBIL). Through the hepatic enzyme UDP-glucuronyl transferase 1A1, IBIL is converted to DBIL. These two kinds of bilirubin subtypes have different clinical implications. Some previous studies have reported the beneficial effects of moderate hyperbilirubinemia only involving TBIL [8] or DBIL [9]. However, it has recently been reported that increasing IBIL is a promising therapeutic approach for treating metabolic diseases [10]. The relationships between serum bilirubins and obesity-related impaired glucose metabolism are still controversial, which may be due to the different types of bilirubin. Indeed, few clinical studies have been conducted on the effects of bilirubin subtypes on insulin sensitivity. Therefore, a clearer appreciation of associations between different types of bilirubin (TBIL, DBIL, and IBIL) and insulin sensitivity will help us to understand the role of bilirubin in obese patients with IGR/T2DM.

Roux-en-Y gastric bypass (RYGB) and sleeve gastrectomy (SG) are the two most common metabolic surgeries worldwide, and they rapidly enhance insulin sensitivity and improve the abnormal blood glucose level even before significant weight reduction [11]. Recent research has found that serum bilirubin can be increased within the physiological range by metabolic surgery [12]. The increase in serum bilirubin levels showed the same trend as the enhancement in insulin sensitivity after metabolic surgery. However, whether the elevated bilirubin levels after metabolic surgery are correlated with an enhancement in insulin sensitivity should be investigated further.

This study aimed to use two cohorts to evaluate the characteristics of various types of bilirubin and their relationships with insulin sensitivity in obese patients with IGR/T2DM in China. Insulin sensitivity of obese patients with or without IGR/T2DM was evaluated using the “gold standard” technique to assess the glucose disposal rate (GDR, *M* value) in cohort 1. As complementary to cohort 1, insulin sensitivity of obese patients with IGR/T2DM was evaluated with the Matsuda Index and homeostatic model assessment of insulin sensitivity (HOMA-IS) in cohort 2. It is hoped that our clinical study provides valuable evidence for subsequent more in-depth basic research on the improvement of obesity-related impaired glucose metabolism.

## 2. Materials and Methods

### 2.1. Participants and Study Design

The study included two independent cohorts. In cohort 1, there were 71 consecutive obese patients (age: 29.85 ± 9.75 years, 41 females) who underwent HEC at the Department of Endocrinology, the First Affiliated Hospital of Nanjing Medical University in Nanjing, China, and they were included between April 1, 2014, to February 28, 2018. Eligible subjects were 16 to 70 years old with a body mass index (BMI) ≧28.0 kg/m^2^ [13], and which were divided into the IGR/T2DM group (*n* *=* 38, obesity with IGR/T2DM) and control group (*n* *=* 33, obesity without IGR/T2DM). T2DM was diagnosed based on fasting plasma glucose (FPG) ≧7.0 mmol/L and/or 2-h plasma glucose (2hPG) ≧11.1 mmol/L and IGR was diagnosed based on 6.1 mmol/L ≦ FPG <7.0 mmol/L and/or 7.8 mmol/L ≦ 2hPG <11.1 mmol/L, according to the World Health Organization (WHO) criteria [14], or having self-reported doctor-diagnosed diabetes or having used antidiabetic agents. The exclusion criteria were as follows: (i) T2DM duration ≧1 year; (ii) having used antidiabetic agents within 3 months; (iii) cardiovascular disease, biliary obstruction disease, acute inflammatory disease, or infectious disease; (iv) serum bilirubin levels ≧2 times upper limit of normal (2ULN) (ULNs : TBIL : 19.0 *μ*mol/L and DBIL : 6.8 *μ*mol/L) or abnormal liver function tests ≧3ULN (ULNs: alanine aminotransferase (ALT) : 40 U/L, aspartate aminotransferase (AST) : 35 U/L, and gamma-glutamyl transferase (GGT) : 45 U/L); (v) smoking or alcohol drinking, or (vi) missing data.

In cohort 2, there were another 109 obese consecutive patients (36.66 ± 12.99 years, 78 females) who underwent RYGB (*n* *=* 87) or SG (*n* *=* 22) at the Department of General Surgery, the First Affiliated Hospital of Nanjing Medical University in Nanjing, China, and they were included between July 1, 2010, to November 30, 2016. Eligible subjects were aged 16–70 years with BMI ≧28.0 kg/m^2^ and IGR/T2DM. The exclusion criteria were consistent with those for cohort 1. Also, subjects who have used antidiabetic agents within 1 month after surgery were ruled out.

This work was conducted with the approval of the Ethical Committee of the First Affiliated Hospital of Nanjing Medical University (2014-SR-003) and was performed according to the Declaration of Helsinki.

Cohort 1 is a case-control study, and insulin sensitivity was evaluated using HEC with glucose disposal rate (GDR, *M* value). Besides, a comprehensive metabolic assessment was performed at baseline. In cohort 2, insulin sensitivity was evaluated using the oral glucose tolerance test (OGTT) with the Matsuda Index and homeostatic model assessment of insulin sensitivity (HOMA-IS). Comprehensive metabolic assessment was performed at baseline and 1 month after surgery.

### 2.2. Insulin Sensitivity Assessment

The OGTT was performed after overnight fasting, and venous blood was obtained at 0, 30, 60, 120, and 180 min after 75 g dextrose administration for measurements of glucose, insulin, and C-peptide concentration.

HEC which was used to evaluate insulin sensitivity was started after overnight fasting of 12 h. Intravenous cannulas were used on different arms for collecting blood samples and intravenous infusion. A warming blanket was used on the blood-collecting arm for the arterialization of venous blood. First, we collected FPG, fasting insulin (FINS), and fasting C-peptide (FC-p); second, on the other side to infused glucose solution and normal human insulin (Humulin R, 40000U/L, Eli Lilly). Through continuous high-dose infusion in the first 10 min, patients rapidly reached the high-concentration insulin state; and insulin was continuously injected at the rate of 2 mU/kg·min in the following 110 min. Blood samples for the measurement of plasma glucose were obtained at 5 min intervals throughout the clamp. A variable infusion of 20% glucose was started to maintain the plasma glucose concentration between 4.5 and 5.5 mmol/L. The glucose disposal rate (GDR, *M* value) was calculated from the glucose infusion rates (GIRs) during the last 30 min of the HEC. The variation coefficient was less than 5% [15].

Insulin sensitivity indices were derived from HEC in cohort 1 and OGTT measurements of glucose and insulin in cohort 2 (Table 1). The cutoff value for insulin resistance was *M* value = 4.9 mg/kg·min [16] in cohort 1.

### 2.3. Clinical Assessment

In the two cohorts, baseline data included the following: gender, age, height in cm, body weight (BW) in kg, and body mass index (BMI) in kg/m^2^. Participants provided a 10–12 h fasting blood sample. Markers of glucose metabolism comprised FPG, FINS, and FC-p; markers of lipid metabolism comprised total cholesterol (TC), triglyceride (TG), high-density lipoprotein cholesterol (HDL-c), and low-density lipoprotein cholesterol (LDL-c); and markers of liver function including alanine aminotransferase (ALT), aspartate aminotransferase (AST), and gamma-glutamyl transferase (GGT) were assessed with a standard clinical automatic analyzer. *M* value, Matsuda Index, and HOMA-IS were calculated. Serum bilirubin levels (TBIL, DBIL, and IBIL) were measured by the oxidation method (Zhuhai Senlong Biotech Co., Ltd., Zhuhai, China) and analyzed using an Olympus AU2700 biochemical analyzer (Olympus Co., Tokyo, Japan).

In cohort 2, postoperative data collection was carried out at 1 month after surgery. Changes were determined by subtracting baseline values from the values at 1 month after surgery. Baseline serum bilirubin levels (BB) were described using TBIL, DBIL, and IBIL at baseline. The percentage change in serum bilirubin levels was described using ∆TBIL%, ∆DBIL%, and ∆IBIL% by subtracting BB from the bilirubin levels at the 1 month follow-up, dividing by BB, and multiplying by 100 [17].

### 2.4. Statistical Analysis

The continuous variables were presented as means ± standard deviations or median (25th/75th percentile). The categorical variables were presented as numbers (%). Differences in normally distributed continuous variables were analyzed using independent samples *t*-test, and nonnormally distributed variables were tested using the Mann–Whitney *U* test. Variables before and after metabolic surgery were compared. Continuous data with normal distribution were compared using the paired sample *t*-test; otherwise, the Wilcoxon test was applied. The chi-squared test was used for comparisons of categorical variables between groups. The correlations between the variables were analyzed using the Spearman correlation coefficient based on the nature of the variables. Multivariate logistic regression analysis was used to analyze the association between different types of bilirubin and insulin resistance; multivariate linear regression was used to analyze the association between postoperative ∆TBIL%, ∆DBIL%, and ∆IBIL% and changes in metabolic variables. Corresponding odds ratios (ORs) and 95% confidence intervals (CIs) were calculated simultaneously. *p* values less than 0.05 were considered significant (two-tailed significance). All statistical analyses were performed using SPSS, version 23.0 (SPSS, IBM, Corp., Armonk, NY, USA).

## 3. Results

### 3.1. Clinical Characteristics of Cohort 1

The basic clinical characteristics of the subjects in cohort 1 are summarized in Table 2. 71 Chinese obese patients (age: 29.85 ± 9.75 years, 41 females) met the eligibility criteria. The patients were divided into the control group (33 patients) and IGR/T2DM group (38 patients). For the patients in cohort 1, serum bilirubin levels (TBIL, DBIL, and IBIL) were higher in the IGR/T2DM group compared with the control group (12.00 (8.90–16.00) vs. 8.80 (7.60–11.60) *μ*mol/L, *p*=0.012; 4.20 (3.20–5.20) vs. 3.10 (2.40–4.20) *μ*mol/L, *p*=0.031; 7.80 (6.10–10.94) vs. 6.20 (4.90–8.10) *μ*mol/L, *p*=0.032)). Insulin sensitivity (*M* value) was lower in the IGR/T2DM group than the control group ((3.73 ± 1.65) vs. (5.01 ± 1.67) mg/kg·min, *p*=0.002)). The differences in BMI, FINS, and FC-p between the two groups were not significant (*p* > 0.05) (Table 2).

### 3.2. Correlations between Serum Bilirubin Levels and Metabolism Variables in Cohort 1

**Figure 1:**
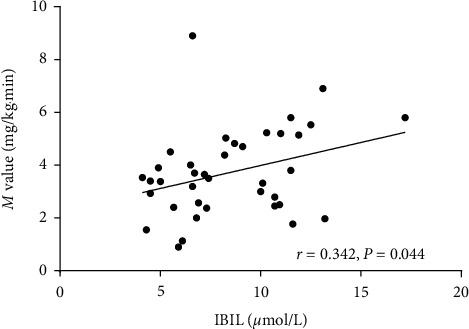
Correlation between *M* value and IBIL of the IGR/T2DM group in cohort 1.The serum IBIL levels positively correlated with *M* value. Abbreviations: *M* value, glucose disposal rate (GDR); IBIL, indirect bilirubin; IGR/T2DM, impaired glucose regulation and type 2 diabetes mellitus.

In the IGR/T2DM group, TBIL was positively correlated with *M* value (*r* *=* 0.325, *p*=0.046) and negatively correlated with FC-p (*r* *=* −0.330, *p*=0.043) but not correlated with other variables; IBIL was positively correlated with *M* value (*r* *=* 0.324, *p*=0.047) and negatively correlated with FINS and FC-p (*r* *=* −0.352, *p*=0.030; *r* *=* -0.349, *p*=0.032) but not correlated with other variables; additionally, there were no correlations between DBIL and any metabolism variables (Table 3). After adjustment for age and gender, the serum TBIL and IBIL levels remained positively correlated with *M* value (*r* *=* 0.344, *p*=0.040; *r* *=* 0.365, *p*=0.028). After further adjustment for age, gender, and BMI, the serum IBIL levels remained positively correlated with *M* value (*r* *=* 0.342, *p*=0.044; Figure 1). There was no correlation between serum bilirubin levels and metabolism variables in the control group (Table 3).

### 3.3. Association between Insulin Resistance and Metabolism Variables of IGR/T2DM Group in Cohort 1

In order to assess the relationship between bilirubin and insulin sensitivity further, the subjects (*n* *=* 38) in the IGR/T2DM group were classified into two categories: patients with or without insulin resistance based on the cutoff value *M* = 4.9 mg/kg·min. In the multivariate logistic regression analysis, it is demonstrated that the higher the bilirubin level, the lower the risk of insulin resistance; TBIL and IBIL might be independent protective factors against insulin resistance (odds ratio (OR) = 0.744, 95% confidence interval (CI) : 0.590–0.938, *p*=0.012; OR = 0.602, 95% CI : 0.413–0.878, *p*=0.008) (Table 4).

### 3.4. Postoperative Changes in Metabolism Variables in Cohort 2

The basic clinical characteristics of the subjects in cohort 2 are summarized in Table 5. Among these subjects, 87 underwent RYGB (79.82%) and 22 underwent SG (20.18%). No postoperative complications or deaths occurred after surgery. There were significant decreases in BW and BMI at 1 month after surgery compared with baseline (all *p* < 0.001, Table 5). The Matsuda Index and HOMA-IS increased significantly (all *p* < 0.001); FPG, FINS, and FC-p decreased significantly (all *p* < 0.001, Table 5) at 1 month after surgery compared with baseline, indicating an enhancement in insulin sensitivity. The serum bilirubin levels (TBIL, DBIL, and IBIL) increased within the physiological range at 1 month after surgery compared with baseline (all *p* < 0.001, Table 5).

### 3.5. Association between Postoperative Percentage Change in Serum Bilirubin Levels and the Changes of Metabolism Variables in Cohort 2

**Figure 2:**
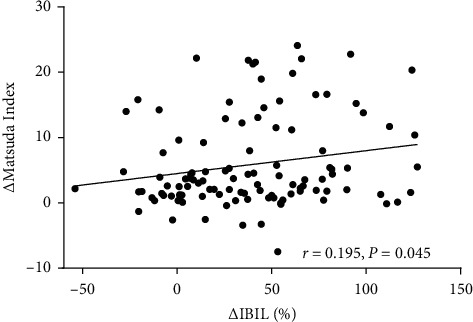
Correlation between ∆Matsuda Index and ∆IBIL% after metabolic surgery in cohort 2. The percentage change in IBIL positively correlated with the change of the Matsuda Index. ∆, difference between selected variable values at one month and preoperatively; IBIL, indirect bilirubin.

At baseline, there were no correlations between serum bilirubin levels and glucose metabolic variables. At 1 month after surgery, the percentage change in TBIL was negatively correlated with the change of FINS and the change of FC-p (*r* *=* −0.217, *p*=0.024; *r* *=* −0.195, *p*=0.042); the percentage change in DBIL was negatively correlated with the change of FINS (*r* *=* −0.192, *p*=0.046); and the percentage change in IBIL was positively correlated with the change of the Matsuda Index and the change of HOMA-IS (*r* *=* 0.195, *p*=0.042; *r* *=* 0.246, *p*=0.010), but it was negatively correlated with the change of FINS and the change of FC-p (*r* *=* −0.235, *p*=0.014; *r* *=* −0.230, *p*=0.016). There were no correlations between the percentage change in serum bilirubin levels (∆TBIL%, ∆DBIL%, and ∆IBIL%) and the changes in other glucose metabolic variables at 1 month after surgery (Table 6). After further adjustment for age, gender, and the change of BMI, the percentage change in IBIL remained positively correlated with the change of the Matsuda Index (*r* *=* 0.195, *p*=0.045; Figure 2). The changes of the Matsuda Index were further analyzed for association with other factors. In multiple linear regression, the best model for prediction of the change of the Matsuda Index after 1 month included the percentage change in IBIL (adjusted *R*^2^ = 0.030), the change of GGT (adjusted *R*^2^ = 0.058), and male (adjusted *R*^2^ = 0.084) (Table 7).

## 4. Discussion

In this study, the characteristics of various types of serum bilirubin levels and their relationships with insulin sensitivity were analyzed in obese patients with or without IGR/T2DM. The cohort 1 data indicated that compared to the simple obese patients, serum bilirubin levels were higher within the physiological range, and IBIL was positively correlated with insulin sensitivity in obese patients with IGR/T2DM. TBIL and IBIL might be independent protective factors against insulin resistance in obese patients with IGR/T2DM.

Traditionally, serum bilirubin has long been considered as an sign of liver dysfunction. Studies have found that higher serum bilirubin levels could decrease the risk of T2DM and serum bilirubin levels were low in diabetes [18, 19]. However, Wang et al. [20] found in the cross-sectional analysis that serum bilirubin levels (total, direct, and indirect) increased in new-onset diabetes. And a longitudinal follow-up study for 4 years found that the bilirubin level was high in patients with new-onset T2DM. Whether the level of bilirubin is low or high in impaired glucose metabolism remains controversial. Our study revealed that serum bilirubin levels were elevated within the physiological range when impaired glucose metabolism occurred in the obese patients, similar to the latter.

The possible mechanism is as follows. The oxidative stress increases during the initial stage of T2DM because the body is exposed to a high-glucose environment. Heme oxygenase-1, the key enzyme for synthesizing bilirubin, may be upregulated to deal with oxidative stress during the stages of prediabetes and new-onset diabetes, and leading to an increase in the downstream product bilirubin so as to play antioxidant effects. However, with the prolonged duration of diabetes, chronic hyperglycemia results in a continuous increase in active oxygen produced by oxidative stress, which leads to an increase in the consumption of bilirubin, and the bilirubin level decrease [20–22]. Whether or not the levels of bilirubin increase might depend on the stage of impaired glucose metabolism. In our study, the participants were young, and they were prediabetes and newly diagnosed with T2DM. They were in the early stage of transition from normal glucose metabolism to abnormal glucose metabolism. Therefore, a compensatory increase in the bilirubin level was observed compared with the level in normal obese patients. We speculate that, with the prolongation of the course of T2DM in our subjects, bilirubin might peak in the early stage of abnormal glucose metabolism and then gradually decrease.

Some studies have focused on the association between bilirubin and insulin resistance [7, 23, 24]; however, few studies or analyses have used HEC, the “gold standard” to evaluate GDR (*M* value) when researched the relationship between serum bilirubin and insulin sensitivity. Our research demonstrated that IBIL were positively correlated with *M* value only in the IGR/T2DM group, but not in the simple obesity group. Their relationships might be affected by the glucose-related metabolic status. A recent study has shown that bilirubin treatment may increase the insulin sensitivity by restraining the endoplasmic reticulum (ER) stress and inflammation of diet-induced obese mice, but the effect is not significant [19]. In case of glucose metabolism abnormality, hyperglycemia may accelerate the physical body to discharge more inflammatory factors, produce more reactive oxygen species (ROS), and intensify ER stress [25, 26]. It is considered that the inhibition effect of bilirubin is amplified with strong ER stress. Bilirubin improves the insulin sensitivity, exerts the function of insulin sensitizer by inhibiting the endoplasmic ER, and improves T2DM. Maybe by this way, it is easily observed that bilirubin is positively correlated with the insulin sensitivity in case of glucose metabolism abnormalities.

By comparing IGR/T2DM patients with or without insulin resistance based on the cutoff value *M* = 4.9 mg/kg·min, our study also showed that, after adjusted the classical factor (including BMI, TC, TG, HDL, and LDL) affecting insulin sensitivity, TBIL and IBIL might be independent protective factors against insulin resistance. The possible mechanisms by which bilirubin may increase insulin sensitivity are as follows. In human and animal studies, bilirubin has been shown to improve insulin resistance by reducing ROS and repairing mitochondrial dysfunction [21, 27, 28]. Meanwhile, bilirubin may enhance insulin sensitivity by decreasing ER stress in the liver [29], suppressing macrophage infiltration in adipose tissue [30], regulating cholesterol metabolism and PPAR-*γ* levels [31], and enhancing insulin signaling pathway [19, 32]. Consequently, mildly elevated serum bilirubin might improve obesity-related dysglycemia by partially enhancing insulin sensitivity.

To further evaluated the relationships between bilirubin and insulin sensitivity in IGR/T2DM patients with obesity, the changes of serum bilirubin and insulin sensitivity were analyzed after metabolic surgery in cohort 2 as complementary to cohort 1. Due to the sample size of patients who underwent HEC assessment of insulin sensitivity before and after surgery is not easy to be acquired, we could not include the same subjects as cohort 1 into cohort 2, so we used the Matsuda Index to assess insulin sensitivity. To minimize selection deviation, we used the same inclusion and exclusion criteria when selecting subjects in both cohorts. The present study demonstrated that compared with baseline, serum bilirubin levels (TBIL, DBIL, and IBIL) increased within the physiological range and insulin sensitivity increased in the short term after surgery. Moreover, a positive correlation was observed between the percentage change in IBIL and the change of insulin sensitivity.

Similar to our results, a study in Saudi Arabia showed that serum total bilirubin was increased slightly in patients who underwent sleeve gastrectomy-induced weight loss during a 1-year follow-up period [12]. Silverman et al. [33] reported there was an increase in total bilirubin from 7.0 mmol/L to 9.6 mmol/L in 91 morbidly obese persons after gastric bypass. These studies demonstrate that serum bilirubin is mildly increased after metabolic surgery. Factors that have an influence on the bilirubin metabolism level include age, sex, smoking, fasting, and other endogenous and external factors. In the fasting, because hemolytic concentration of bilirubin in enterohepatic and intestinal areas is elevated, the level of serum bilirubin is also increased [34]. After the metabolic surgery, the gastric volume of patients decreases partially in similarity of fasting. This may be one of the reasons to cause elevated serum bilirubin, whereas the obesity is considered a lower-level inflammatory state that increases the lipid peroxidation and active oxygen to restrain the antioxidant cleaning mechanism [12]. After the metabolic surgery, oxidative stress response and chronic inflammation decrease, which declines the consumption of bilirubin as the antioxidant [12, 32]. In addition, the mechanisms that affect bilirubin metabolism after metabolic surgery might be related to anatomical intestinal changes and changes in gastrointestinal hormones, intestinal flora, and bile acid metabolism [35–37]. In our study, we focus on the characteristics of changes in serum bilirubin after metabolic surgical intervention, and the possible external factors that interfere with the level of bilirubin have been excluded, except for the surgical operation.

Multiple linear regression analysis showed that the percentage change in IBIL might independently influence the change of the Matsuda Index. This suggests that slightly elevated bilirubin might enhance insulin sensitivity independent of classical factors. Bilirubin can be increased in the short term after metabolic surgery, and the elevation of IBIL might play a partial role in enhancing insulin sensitivity. However, due to lack of larger sample size and more longitudinal follow-up, the interpretation is limited.

Combining the results from the two independent cohorts, our study shows that the relationships between different components of bilirubin (TBIL, DBIL, and IBIL) and insulin sensitivity varied; IBIL, which is the main component of TBIL, might play a major role in enhancing insulin sensitivity. Bulmer et al. [38] reported the risk of cardiovascular diseases is low in patients with Gilbert's syndrome who have congenital and physiological IBIL elevation. Inoguchi et al. [39] demonstrated that the risk of vascular complications is lower in diabetes patients with Gilbert's syndrome. In our study, the effect of the increase in IBIL (within the physiological range) on obese patients with IGR/T2DM may be similar to the effect in patients with Gilbert's syndrome. IBIL is one of the most potent endogenous antioxidant substances, so it can decrease the oxidative stress in cells. In addition, IBIL and the UGT1A1*∗*28 genotype indirectly influence glucose-related metabolic status, as they exert direct upstream effects on factors involved in the energy metabolism, including AMP-activated protein kinase (AMPK) and PPAR-*α* [34, 40]. It has been proposed that IBIL is a promising therapeutic agent for obesity and T2DM [41, 42].Moreover, atazanavir (which increases IBIL) is considered to improve endothelium-dependent relaxation of blood vessels in patients with diabetes [43]. Thus, we hypothesize that IBIL might be a protective factor that enhances insulin sensitivity and serve as an insulin sensitizer, delaying the occurrence and development of metabolic diseases.

Certainly, several limitations should be acknowledged here. First of all, in cohort 1, because of the complexity of HEC operation technology, the sample size for repeated measurement is limited; in cohort 2, the sample size for HEC assessment of insulin sensitivity before and after metabolic surgery is not easy to obtain, so it cannot be included in the same subjects as cohort 1, and the subjects from the IGR/T2DM group in cohort 1 are different from those in cohort 2. In order to minimize selection deviation, we used the same inclusion and exclusion criteria in the two cohorts. Second, only the 1-month follow-up after metabolic surgery had relatively complete data and only preliminary conclusions were reached. Furthermore reasonable scientific design and large prospective population-based studies with long follow-up durations are needed to confirm the effects of bilirubin on insulin sensitivity. Mild elevations of bilirubin can result in clinical benefits, and they are expected to have a big therapeutic potentiality. It is hoped that our study provides clinical evidence to guide further basic research on obesity-related impaired glucose metabolism.

## 5. Conclusions

In summary, our study demonstrated that in Chinese obese patients with IGR/T2DM, the higher the indirect bilirubin within the physiological range, the higher the insulin sensitivity, the greater the increase of the percentage change in indirect bilirubin 1 month after metabolic surgery, and the greater the enhancement in insulin sensitivity. Serum indirect bilirubin, which is the main component of total bilirubin, might be a protective factor that enhances insulin sensitivity.

## Figures and Tables

**Table 1:** Insulin sensitivity indices derived from HEC and OGTT measurements of glucose and insulin.

Index	Formula/equation
*M* value (mg/kg·min)	GIR + (G_a_-G_a-30_) × 0.0625
Matsuda Index	10000/(FPG × FINS × G_mean_ × I_mean_)^1/2^
HOMA-IS = (1/HOMA-IR)	1/[FPG (mmol/L) × FINS (mIU/L)/22.5]

*M* value, glucose disposal rate (GDR); GIR (mg/kg·min), the glucose infusion rate was measured every 5 min, and the average glucose infusion rate was measured during the final 30 min of HEC; G_a_, plasma glucose concentration (mmol/L) at 120 minutes of HEC; G_a-30_, plasma glucose concentration (mmol/L) at 90 minutes of HEC; Matsuda Index, insulin sensitivity index by Matsuda; G_mean_, mean of plasma glucose concentration (mmol/L) during the OGTT; I_mean_, mean of serum insulin concentration (mIU/L) during the OGTT. Abbreviations: HEC, hyperinsulinemic-euglycemic clamp; OGTT, oral glucose tolerance test; HOMA-IS, homeostasis model assessment of insulin sensitivity; HOMA-IR, homeostasis model assessment of insulin resistance; FPG, fasting plasma glucose; FINS, fasting insulin.

**Table 2:** Clinical and metabolic characteristics of the two subgroups at baseline in cohort 1.

	Control group	IGR/T2DM group	*t/Z/χ* ^*2*^	*p* value
	(*n* = 33)	(*n* = 38)		
Age (years)	25.33.±7.16	33.68.±10.22	3.909	<0.001
Female, *n* (%)	11 (33.33%)	19 (50.00%)	2.011	0.156
BW (kg)	98.60 (86.90–112.00)	98.40 (87.50–112.00)	−0.046	0.963
BMI (kg/m^2^)	36.29 (32.27–39.00)	34.05 (31.01–41.06)	−0.380	0.704
FPG (mmol/L)	4.81 (4.50–5.32)	6.39 (5.20–7.73)	−4.514	<0.001
FINS (mU/L)	28.12 (21.36–30.10)	31.44 (21.46–36.52)	−0.749	0.454
FC-p (pmol/L)	1375.28 ± 390.79	1410.27 ± 457.99	−0.343	0.732
M value (mg/kg·min)	5.01 ± 1.67	3.73 ± 1.65	−3.251	0.002
TBIL (*μ*mol/L)	8.80 (7.60–11.60)	12.00 (8.90–16.00)	−2.513	0.012
DBIL (*μ*mol/L)	3.10 (2.40–4.20)	4.20 (3.20–5.20)	−2.151	0.031
IBIL (*μ*mol/L)	6.20 (4.90–8.10)	7.80 (6.10–10.94)	−2.139	0.032
TC (mmol/L)	4.82 ± 0.98	5.04 ± 1.12	0.878	0.383
TG (mmol/L)	1.26 (0.86–1.69)	1.72 (1.27–2.41)	−2.081	0.037
HDL-c (mmol/L)	1.14 ± 0.17	1.11 ± 0.25	−0.612	0.543
LDL-c (mmol/L)	3.17 (2.67–3.56)	3.23 (2.52–3.85)	−0.386	0.699
ALT (U/L)	30.80 (19.00–68.70)	59.10 (26.20–78.60)	−1.591	0.112
AST (U/L)	26.60 (18.90–38.00)	38.25 (20.20–47.60)	−1.470	0.142
GGT (U/L)	31.00 (21.40–49.50)	42.60 (34.70–66.30)	−2.519	0.012

Data are shown as mean ± SD for normally distributed variables or as median (25^th^ and 75th percentiles) or *n* (%), and *p* < 0.05 is considered statistically significant. Abbreviations: IGR/T2DM, impaired glucose regulation and type 2 diabetes mellitus; BW, body weight; BMI, body mass index; FPG, fasting plasma glucose; FINS, fasting insulin; FC-p, fasting C-peptide; *M* value, glucose disposal rate (GDR); TBIL, total bilirubin; DBIL, direct bilirubin; IBIL, indirect bilirubin; TC, total cholesterol; TG, triglyceride; HDL-c, high-density lipoprotein cholesterol; LDL-c, low-density lipoprotein cholesterol; ALT, alanine transaminase; AST, aspartate aminotransferase; GGT, gamma-glutamyl transferase.

**Table 3:** Correlations between serum bilirubin levels (TBIL, DBIL, and IBIL) and metabolism variables of the two subgroups in cohort 1.

	Control group						IGR/T2DM group					
	TBIL (*μ*mol/L)		DBIL (*μ*mol/L)		IBIL (*μ*mol/L)		TBIL (*μ*mol/L)		DBIL (*μ*mol/L)		IBIL (*μ*mol/L)	
	*r*	*p* value	*r*	*p* value	*r*	*p* value	*r*	*p* value	*r*	*p* value	r	*p* value
Weight (kg)	−0.215	0.229	−0.276	0.120	−0.042	0.816	−0.266	0.106	−0.207	0.213	−0.259	0.116
BMI (kg/m^2^)	−0.195	0.276	−0.321	0.069	0.031	0.862	−0.294	0.073	−0.257	.0119	−0.293	0.074
FPG (mmol/L)	0.039	0.830	−0.003	0.986	−0.033	0.857	−0.030	0.860	−0.123	0.462	0.027	0.873
FINS (mU/L)	0.047	0.794	0.127	0.481	0.082	0.651	−0.317	0.052	−0.289	0.078	−**0.352**	**0.030**
FC-p (pmol/L)	−0.123	0.496	−0.028	0.877	−0.022	0.902	−**0.330**	**0.043**	−0.273	0.097	−**0.349**	**0.032**
M value (mg/kg·min)	0.078	0.666	0.249	0.163	−0.026	0.884	**0.325**	**0.046**	0.319	0.051	**0.324**	**0.047**
ALT (U/L)	0.237	0.184	**0.356**	**0.042**	0.123	0.497	0.285	0.083	0.323	0.048	0.254	0.124
AST (U/L)	0.235	0.189	0.268	0.131	0.145	0.420	0.211	0.204	0.194	0.243	0.200	0.228
ALP (U/L)	0.161	0.371	0.154	0.392	0.090	0.618	−0.001	0.994	0.012	0.943	0.018	0.915
GGT (U/L)	0.183	0.308	**0.347**	**0.048**	0.106	0.557	0.077	0.647	0.083	0.621	0.057	0.734
TC (mmol/L)	−0.126	0.484	−0.138	0.443	−0.070	0.698	0.011	0.950	−0.251	0.128	0.099	0.556
TG (mmol/L)	−0.100	0.578	−0.166	0.356	0.002	0.992	−0.285	0.083	−0.269	0.103	−0.278	0.091
HDL-c (mmol/L)	0.034	0.849	−0.116	0.521	−0.058	0.750	0.063	0.706	−0.080	0.634	0.147	0.379
LDL-c (mmol/L)	−0.017	0.927	−0.040	0.826	0.014	0.938	0.023	0.889	−0.123	0.463	0.031	0.855

IGR/T2DM, impaired glucose regulation and type 2 diabetes mellitus; BW, body weight; BMI, body mass index; FPG, fasting plasma glucose; FINS, fasting insulin; FC-p, fasting C-peptide; *M* value, glucose disposal rate (GDR); ALT, alanine transaminase; AST, aspartate aminotransferase; GGT, gamma-glutamyl transferase; TC, total cholesterol; TG, triglyceride; HDL-c, high-density lipoprotein cholesterol; LDL-c, low-density lipoprotein cholesterol; TBIL, total bilirubin; DBIL, direct bilirubin; IBIL, indirect bilirubin. Spearman *r* correlation, *p* < 0.05. Statistically significant values are indicated in bold.

**Table 4:** Multivariate logistic regression analysis, using a cutoff value for insulin resistance of the IGR/T2DM group, after adjustment for metabolism variables in cohort 1.

	Model 1		Model 2		Model 3	
	OR (95% CI)	*p* value	OR (95% CI)	*p* value	OR (95% CI)	*p* value
TBIL (*μ*mol/L)	0.744 (0.590–0.938)	0.012	0.744 (0.590–0.938)	0.012	0.744 (0.590–0.938)	0.012
DBIL (*μ*mol/L)	0.575 (0.326–1.015)	0.056	0.575 (0.326–1.015)	0.056	0.575 (0.326–1.015)	0.056
IBIL (*μ*mol/L)	0.602 (0.413–0.878)	0.008	0.602 (0.413–0.878)	0.008	0.602 (0.413–0.878)	0.008

IGR/T2DM, impaired glucose regulation and type 2 diabetes mellitus; SE, standard error; Exp (B), odds ratio; CI, confidence interval; TBIL, total bilirubin; DBIL, direct bilirubin; IBIL, indirect bilirubin; BMI, body mass index; TC, total cholesterol; TG, triglyceride; HDL-c, high-density lipoprotein cholesterol; LDL-c, low-density lipoprotein cholesterol; ALT, alanine transaminase; AST, aspartate aminotransferase; GGT, gamma-glutamyl transferase. Multivariate logistic regression analysis, *p* < 0.05. Statistically significant values are indicated in bold. Model 1: adjusted for age and sex; Model 2: additionally adjusted for BMI, TC, TG, HDL, and LDL; Model 3: additionally adjusted for ALT, AST, and GGT.

**Table 5:** Metabolic changes after metabolic surgery in cohort 2 (*n* = 109).

Variable	Baseline	1 month	*t/Z*	*p* value
BW (kg)	105.83 ± 22.01	92.35 ± 19.20	17.925	<0.001
BMI (kg/m^2^)	38.44 ± 6.87	33.55 ± 6.12	18.957	<0.001
FPG (mmol/L)	6.80 (5.46–8.96)	5.24 (4.73–6.40)	−6.681	<0.001
FINS (mU/L)	21.72 (9.68–30.08)	9.29 (2.20–14.87)	−7.552	<0.001
FC-p (pmol/L)	1324.66 ± 619.81	834.39 ± 496.48	8.556	<0.001
Matsuda Index	1.80 (1.25–4.12)	5.38 (3.03–14.91)	−8.092	<0.001
HOMA-IS	0.16 (0.10–0.33)	0.43 (0.25–1.70)	−7.690	<0.001
TBIL (*μ*mol/L)	9.60 (7.87–11.70)	13.37 (11.65–15.80)	−8.176	<0.001
DBIL (*μ*mol/L)	3.60 (2.67–4.40)	5.20 (4.60–6.14)	−7.614	<0.001
IBIL (*μ*mol/L)	6.03 (4.90–7.43)	8.20 (6.60–10.05)	−7.200	<0.001
ALT (U/L)	28.00 (17.60–48.60)	32.20 (16.80–55.90)	−0.432	0.666
AST (U/L)	23.30 (16.00–34.30)	33.10 (23.20–46.30)	−4.576	<0.001
GGT (U/L)	29.20 (19.80–46.40)	23.23 (15.60–31.15)	−4.022	<0.001
TC (mmol/L)	4.80 (4.16–5.41)	4.20 (3.60–4.64)	−6.323	<0.001
TG (mmol/L)	1.47 (1.21–2.29)	1.28 (1.10–1.55)	−4.602	<0.001
HDL-c (mmol/L)	1.11 (0.89–1.27)	0.91 (0.83–1.17)	−4.587	<0.001
LDL-c (mmol/L)	3.19 ± 0.70	2.88 ± 0.71	4.454	<0.001

Data are shown as mean ± SD for normally distributed variables or as median (25^th^ and 75^th^ percentiles), and *p* < 0.05 is considered statistically significant. BW, body weight; BMI, body mass index; FPG, fasting plasma glucose; FINS, fasting insulin; FC-p, fasting C-peptide; HOMA-IS: homeostasis model assessment of insulin sensitivity; TBIL, total bilirubin; DBIL, direct bilirubin; IBIL, indirect bilirubin; TC, total cholesterol; TG, triglyceride; HDL-c, high-density lipoprotein cholesterol; LDL-c, low-density lipoprotein cholesterol; ALT, alanine transaminase; AST, aspartate aminotransferase; GGT, gamma-glutamyl transferase.

**Table 6:** Correlations between the postoperative percentage change in serum bilirubin levels (∆TBIL%, ∆DBIL%, and ∆IBIL%) and the changes of metabolism variables in cohort 2.

	∆TBIL%		∆DBIL%		∆IBIL%	
	*r*	*p* value	*r*	*p* value	*r*	*p* value
∆BMI (kg/m^2^)	−0.170	0.077	−0.185	0.054	−0.136	0.157
∆FPG (mmol/L)	−0.071	0.463	0.012	0.901	−0.159	0.099
∆FINS (mU/L)	**−0.217**	**0.024**	**−0.192**	**0.046**	**−0.235**	**0.014**
∆ FC-p (pmol/L)	**−0.195**	**0.042**	−0.128	0.184	**−0.230**	**0.016**
∆Matsuda Index	0.062	0.522	−0.074	0.447	**0.195**	**0.042**
∆HOMA-IS	0.100	0.303	0.056	0.563	**0.246**	**0.010**
∆ ALT (U/L)	−0.046	0.632	−0.065	0.504	−0.006	0.948
∆ AST (U/L)	0.033	0.731	−0.029	0.766	0.087	0.367
∆ GGT (U/L)	0.150	0.120	0.077	0.426	0.171	0.076
∆ TC (mmol/L)	0.007	0.941	−0.072	0.455	0.065	0.499
∆ TG (mmol/L)	0.020	0.840	0.029	0.767	0.012	0.898
∆ HDL-c (mmol/L)	0.002	0.986	−0.062	0.520	0.029	0.765
∆ LDL-c (mmol/L)	0.089	0.357	0.034	0.725	0.125	0.194

∆, difference between selected variable values at one month and preoperatively; BMI, body mass index; FPG, fasting plasma glucose; FINS, fasting insulin; FC-p, fasting C-peptide; HOMA-IS: homeostasis model assessment of insulin sensitivity; ALT, alanine transaminase; AST, aspartate aminotransferase; GGT, gamma-glutamyl transferase; TC, total cholesterol; TG, triglyceride; HDL-c, high-density lipoprotein cholesterol; LDL-c, low-density lipoprotein cholesterol; TBIL, total bilirubin; DBIL, direct bilirubin; IBIL, indirect bilirubin. Spearman *r* correlation, *p* < 0.05. Statistically significant values are indicated in bold.

**Table 7:** Multivariate linear regression analysis between the changes of the Matsuda Index and the changes of variable postoperative alterations in cohort 2.

Dependent variable	Independent variable	Standardized *β*	SE	*p* value	Adjusted *R* square
∆Matsuda Index	∆IBIL%	0.217	0.017	0.022	0.030
	∆ GGT	−0.208	0.025	0.028	0.058
	Male	−0.184	1.425	0.049	0.084

∆, difference between selected variable values at one month and preoperatively; BMI, body mass index; GGT, gamma-glutamyl transferase. Multivariate linear regression analysis, *p* < 0.05. Statistically significant values are indicated in bold.

## Data Availability

The data used and/or analyzed during the current study are available from the corresponding author on reasonable request.
